# Optogenetic regulation of insulin secretion in pancreatic β-cells

**DOI:** 10.1038/s41598-017-09937-0

**Published:** 2017-08-24

**Authors:** Fan Zhang, Emmanuel S. Tzanakakis

**Affiliations:** 1 Department of Chemical and Biological Engineering, Tufts University, Medford, MA 02155 USA; 2 Clinical and Translational Science Institute, Tufts Medical Center, Boston, MA 02111 USA

## Abstract

Pancreatic β-cell insulin production is orchestrated by a complex circuitry involving intracellular elements including cyclic AMP (cAMP). Tackling aberrations in glucose-stimulated insulin release such as in diabetes with pharmacological agents, which boost the secretory capacity of β-cells, is linked to adverse side effects. We hypothesized that a photoactivatable adenylyl cyclase (PAC) can be employed to modulate cAMP in β-cells with light thereby enhancing insulin secretion. To that end, the PAC gene from *Beggiatoa* (bPAC) was delivered to β-cells. A cAMP increase was noted within 5 minutes of photostimulation and a significant drop at 12 minutes post-illumination. The concomitant augmented insulin secretion was comparable to that from β-cells treated with secretagogues. Greater insulin release was also observed over repeated cycles of photoinduction without adverse effects on viability and proliferation. Furthermore, the expression and activation of bPAC increased cAMP and insulin secretion in murine islets and in β-cell pseudoislets, which displayed a more pronounced light-triggered hormone secretion compared to that of β-cell monolayers. Calcium channel blocking curtailed the enhanced insulin response due to bPAC activity. This optogenetic system with modulation of cAMP and insulin release can be employed for the study of β-cell function and for enabling new therapeutic modalities for diabetes.

## Introduction

Precise control of complex cellular functions with external stimuli is essential for engineering effective cell therapeutics. Pharmacological manipulations typically exhibit poor cellular specificity and temporal control that is not harmonized with the timescale of relevant physiological processes. One such function is the glucose-stimulated insulin secretion (GSIS) by pancreatic β-cells that is central to blood glucose homeostasis. Aberrant insulin production is a hallmark of diabetes resulting from autoimmune destruction of β-cells (type 1 diabetes; T1D) or hormone resistance by tissues absorbing glucose (type 2 diabetes; T2D). GSIS in β-cells starts with the metabolism of glucose and the ATP/ADP-dependent closure of ATP-sensitive K^+^ (K_ATP_) channels resulting in membrane depolarization and opening of the voltage-gated Ca^2+^ channels^1^. The influx of Ca^2+^ and increase of its concentration ([Ca^2+^]_i_) elicit exocytosis of insulin secretory granules. Of particular relevance to T2D treatment, hormone release can be boosted with secretagogues acting on intermediates of the insulin secretion circuitry in β-cells. Nonetheless, the lack of specificity in such treatments diminishes their effectiveness. For instance, sulfonylureas trigger the closure K^+^_ATP_ channels in β-cells and the ensuing membrane depolarization causes insulin secretion regardless of plasma glucose concentrations increasing the risk for hypoglycemic episodes^2^. K^+^_ATP_ channels are also found in other cell types (e.g. cardiomyocytes, non-vascular smooth muscle cells) making such treatments prone to additional side effects^3^.

To that end, optogenetic approaches have been employed for drug-free control with light of processes including neuronal cell activity^4^, contractility of cardiomyocytes^5^ and skeletal muscle cells^6^, and depolarization of retinal ganglion cells^7^. These strategies entail the creation of synthetic cellular circuits with light-activated molecules for the manipulation of signaling moieties thereby providing a handle on relevant functions. Optogenetic regulation of glucose homeostasis has been reported with the expression of bacterial channelrhodopsins (ChRs), which respond to light by inducing fluxes of specific ions. Human embryonic kidney 293 (HEK293) cells engineered to display melanopsin, expressed glucagon-like peptide-1 (GLP-1) from an endogenous factor of activated T cells (NFAT)-responsive promoter upon stimulation with blue light^8^. A return to normoglycemia was noted in diabetic mice after subcutaneous implantation of the engineered HEK293 cells. Along the same vein, others demonstrated the optogenetic control of Ca^2+^ influx in β-cells with the expression of ChRs^9, 10^. These results illustrate the feasibility of implementing optogenetic approaches to regulate blood glucose homeostasis. Nevertheless, the light- or agent-induced (e.g. by ionomycin^11^) increases in [Ca^2+^]_i_ can lead to insulin secretion by β-cells in the absence of glucose pointing to the inherent risk imposed by ChR-based systems for hypoglycemic excursions.

Cyclic AMP (cAMP) is a major regulator^12, 13^ of GSIS through its effects on protein kinase A (PKA), the exchange protein activated by cAMP (Epac), and the recruitment of insulin vesicles and their secretion^14^. Intracellular cAMP ([cAMP]_i_) is synthesized from ATP by adenylyl cyclases (ACs) while phosphodiesterases (PDEs) are tasked with its rapid degradation. Consequently, AC activation (e.g. by forskolin) or PDE inhibition (e.g. by 3-isobutyl-1-methylxanthine; IBMX) augments GSIS. Incretins such as the GLP-1 and glucose-dependent insulinotropic polypeptide (GIP) released by intestinal cells elevate cAMP in islet β-cells to reduce postprandial blood glucose. While cAMP is an intracellular amplifier of GSIS, it does not induce the release of insulin in the absence of glucose in contrast to [Ca^2+^]_i_. As such, cAMP is an attractive target for boosting insulin production particularly in diabetes therapies^15–17^.

To that end, manipulation of cAMP using light has been demonstrated in *Drosophila* cells, *Xenopus* oocytes and HEK293 cells^18^ heterologously expressing photoactivatable ACs (PACs) from lower organisms^19, 20^. In this study, we hypothesized that β-cell insulin secretion can be controlled by modulating [cAMP]_i_ with illumination. For this purpose, a PAC from the bacterium *Beggiatoa* (bPAC)^20, 21^ was expressed *in vitro* in murine islets and in β-cell lines, which closely mirror the functional attributes of native β-cells. We show that irradiation of bPAC expressed in β-cells elevates [cAMP]_i_ enhancing insulin release, even over repeated cycles of stimulation with blue light. This augmented insulin secretion, which was evident both in monolayers and pseudoislets (PIs) of bPAC-expressing β-cells, was abolished in the presence of a Ca^2+^ channel blocker, indicating its dependence on Ca^2+^ influx in the same fashion as a typical GSIS response in islet cells.

## Results

### Expression of bPAC and [cAMP]_i_ modulation in MIN6 β-cells

**Figure 1:**
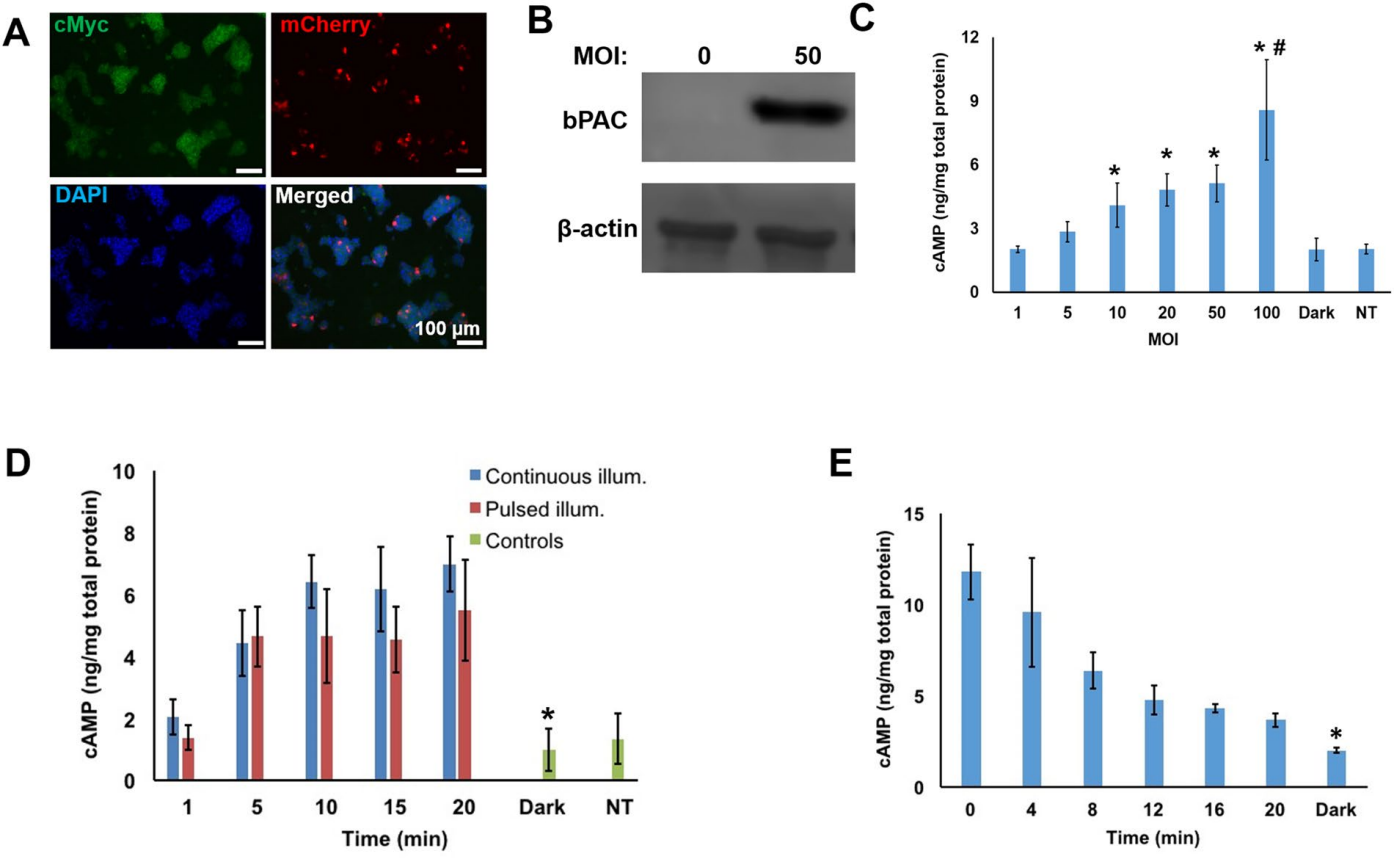
Expression of bPAC confers light-induced modulation of [cAMP]_i_ in MIN6 β-cells. (**A**) Fluorescence micrographs of AdbPAC-transduced cells (MOI = 100) stained for bPAC (antibody against the myc tag) and nuclear DNA (DAPI). Expressed mCherry (epifluorescence) is also shown. Bar: 100 µm. (**B**) Western blot showing the expression of bPAC (myc tag antibody) and β-actin. [cAMP]_i_ levels are shown for various (**C**) MOI and (**D**) durations of pulsed (red bars) or continuous illumination (blue bars). Cells in (**C**) were illuminated for 10 min continuously. *p < 0.05 vs. dark, ^#^p < 0.05 vs. cells infected at MOI of 10–50. Cells in (**D**) were infected at MOI = 100. *p < 0.05 vs. illumination for 5–20 min. In (**C**,**D**): NT: Non-transduced cells. (**E**) [cAMP]_i_ levels after 10 min of illumination. Times after termination of the illumination are shown. *p < 0.05 vs. samples at 12–20 min. In (**C**–**E**): Dark: AdbPAC-infected cells (MOI = 100) without illumination. Results are shown as mean ± SD from at least three independent experiments, with each measurement carried out in triplicates. Some blots were edited for better (clear cut) representation. The entire blots are included in Supplementary Materials.

We set out to investigate if [cAMP]_i_ and insulin release can be modulated via light in β-cells upon expression of the bPAC from *Beggiatoa*. An adenoviral vector, AdbPAC, was constructed for delivering the human codon-optimized bPAC to MIN6 β-cells. The myc tag (fused to the C-terminus of bPAC) and fluorescent protein mCherry were included in the cassette to facilitate the detection of bPAC (Fig. 1A,B). Based on immunostaining and fluorescence, the expression of mCherry was seemingly lower (Fig. 1A), which is typical for cistrons downstream of an IRES element^22, 23^. Indeed, the fraction of cells expressing bPAC was determined at different multiplicities of infection (MOI) either by immunostaining for the myc tag and microscopy or by flow cytometry for mCherry (Suppl. Fig. S1). Transduction levels increased to almost 55% for MOI of 100 with greater than 70% cell viability. When [cAMP]_i_ was measured, we noted an increase that was commensurate with the bPAC expression based on the MOI (Fig. 1C). Cells infected at MOI of 100 displayed the highest [cAMP]_i_ level with a 10-min photoinduction (8.59 ± 2.36 vs. 2.03 ± 0.53 (dark) ng cAMP/mg total protein, p = 0.035, n = 3).

We also probed the relationship between the duration of illumination and increase in [cAMP]_i_ (‘on’ kinetics). For this purpose, cultures transduced with AdbPAC were stimulated with continuous or pulsed light (5 s on/10 s off) for various durations between 1–20 min in 2.5 mM glucose (Fig. 1D). Compared to bPAC-expressing cells kept in dark, cells exposed to blue light for 5 min or longer exhibited a 6- to 8-fold increase in [cAMP]_i_. There was also no difference between the two modes of photostimulation in this setup. Conversely, the (‘off’) kinetics were determined of [cAMP]_i_ reduction post-illumination (Fig. 1E). After a 10-min illumination, [cAMP]_i_ levels returned to a stable baseline within 12 min albeit higher than for cells kept in dark (4.75 ± 0.78 vs. 1.99 ± 0.14 ng cAMP/mg total protein, p = 0.023, n = 3). Incubation of bPAC-expressing MIN6 β-cells with an AC inhibitor led to a reduction in the [cAMP]_i_ (Suppl. Fig. S2) further illustrating that the observed increase in the secondary messenger is due to the action of bPAC upon photoactivation. Taken together, our data show that heterologous expression of bPAC in β-cells allows the on-demand alteration of [cAMP]_i_ with light following physiologically relevant kinetics similar to [cAMP]_i_ changes in islet β-cells exposed to changing concentrations of glucose^24^.

### Insulin secretion in β-cells expressing bPAC

**Figure 2:**
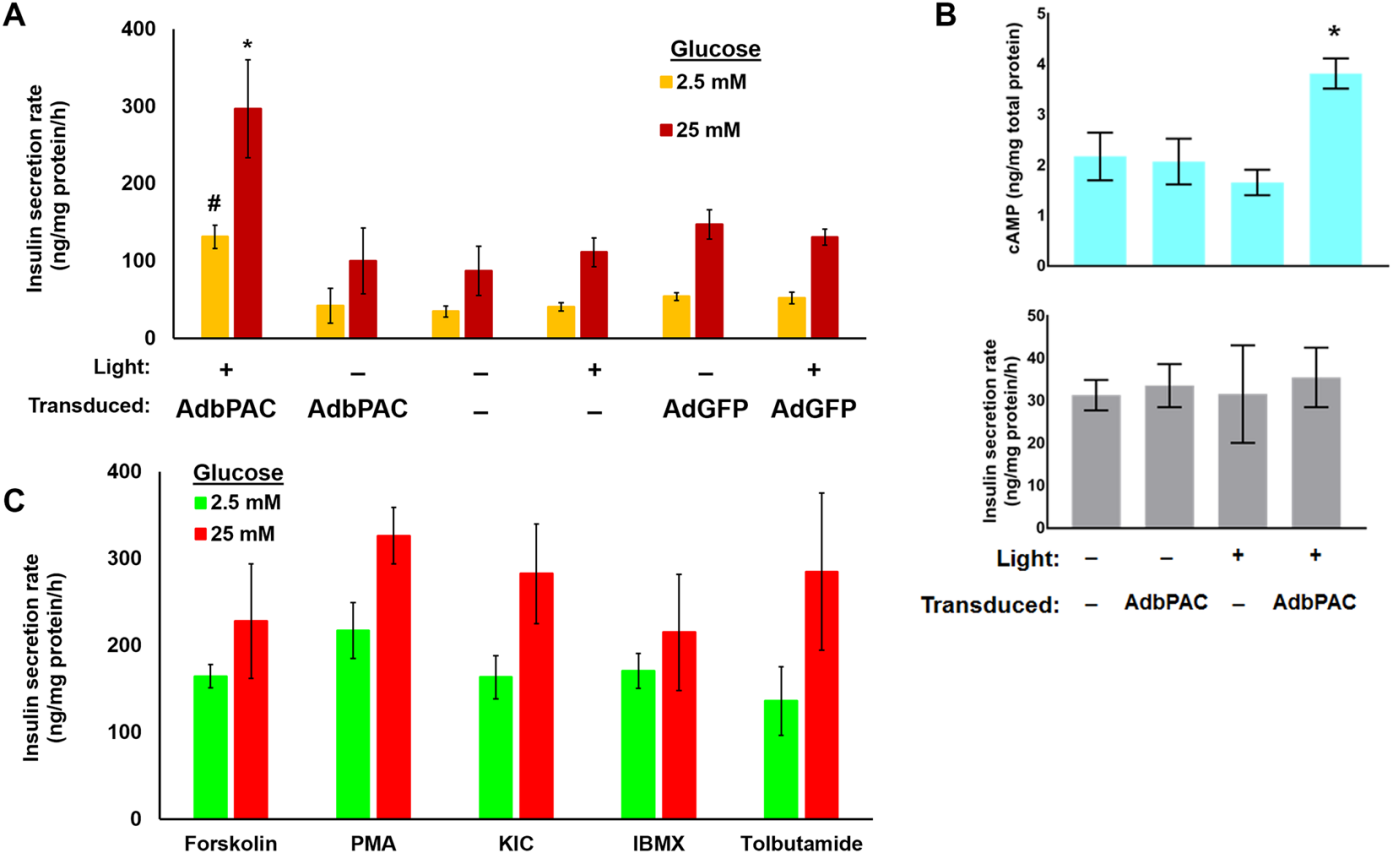
Insulin secretion by β-cells expressing bPAC. (**A**) MIN6 β-cells expressing bPAC displayed higher rates of insulin release (ng insulin/mg total protein/h) upon exposure to light compared to cells kept in dark and non-transduced cells or cells infected with AdGFP. MOI = 100; illumination for 30 min; *p < 0.05 vs. all other samples at 25 mM glucose; ^#^p < 0.05 vs. all other samples at 2.5 mM glucose. (**B**) [cAMP]_i_ and insulin secretion for MIN6 β-cells in the absence of glucose (MOI = 100); *p < 0.05 vs. all other samples. (**C**) Insulin secretion was determined in MIN6 β-cell monolayers (without bPAC expression) treated with various secretagogues at 2.5 or 25 mM glucose (see Materials and Methods). Results are shown as mean ± SD from at least three independent experiments.

The light-mediated modulation of [cAMP]_i_ in bPAC-expressing β-cells prompted us to investigate if insulin release could be regulated in an analogous manner. AdbPAC-transduced MIN6 β-cells at 2.5 mM of glucose secreted 131.65 ± 15.14 ng insulin/mg total protein/h (Fig. 2A) while the rate for non-transduced MIN6 β-cells was 34.97 ± 6.99 ng insulin/mg total protein/h (p = 2.74 × 10^−3^, n = 4). Similarly, bPAC-expressing cells at 25 mM glucose released 296.6 ± 63.25 vs. 87.57 ± 31.8 ng insulin/mg total protein/h (p = 6.91 × 10^−3^, n = 4) for control cells. Thus, blue light triggered an analogous increase (~3.5-fold) in bPAC-positive cells at low and high concentrations of glucose. In contrast, this increase was not observed in bPAC-expressing cells that were kept in dark. Of note, cells infected with AdGFP (control) did not differ in their hormone secretion from uninfected cells, suggesting that the adenoviral transduction did not affect the secretory function of β-cells. Additionally, the expression of bPAC and photostimulation augmented the rate of insulin release in another β-cell line (βTC cells; Suppl. Fig. S3), supporting the notion that the enhanced insulin secretion that we observed upon photoexposure of bPAC was not applicable only to MIN6 β-cells. The ratio of hormone secretion rates at 25 mM to 2.5 mM glucose was similar (~2.2–2.5) between cells expressing bPAC and those that did not suggesting that light-stimulated bPAC causes a surge in the rate of insulin release but at a constant ratio.

These data show that elevation of [cAMP]_i_ amplifies the secretion of insulin in the presence of glucose, even at 2.5 mM glucose, in bPAC-expressing MIN6 β-cells after activation with light. This behavior parallels the response of AdbPAC-transduced primary islets (see results in the next section) after photostimulation and is in support of a previous report indicating the resemblance in the regulation of insulin secretion between particular insulinoma lines and normal β-cells^25^.

Most transformed β-cell lines however exhibit hypersensitivity to glucose compared to primary β-cells^26^. This prompted us to measure the [cAMP]_i_ and insulin secretion rate in the absence of glucose. Indeed, while AdbPAC-transduced MIN6 β-cells exhibited a significantly higher [cAMP]_i_ after illumination (3.82 ± 0.3 vs. 2.17 ± 0.47 ng /mg total protein for cells without transduction; p = 3.3 × 10^−3^, n = 3), their insulin secretion was not different than that of the control cultures (Fig. 2B).

We also compared the enhanced hormone release due to treatment with blue light of bPAC-expressing cells with that resulting from incubation with various secretagogues (Fig. 2C). The responses were statistically similar even when cells were cultured with forskolin (AC activator) and IBMX (phosphodiesterase inhibitor), indicating that bPAC expression and activation by light mimics the amplifying effects of secretagogues on the insulin release by β-cells. Of note, the [cAMP]_i_ levels of AdbPAC-transduced cells exposed to light were higher than for β-cells treated with forskolin or IBMX (Suppl. Fig. S4), suggesting that there may be an upper threshold in [cAMP]_i_ above which there is no further significant increase in insulin secretion.

**Figure 3:**
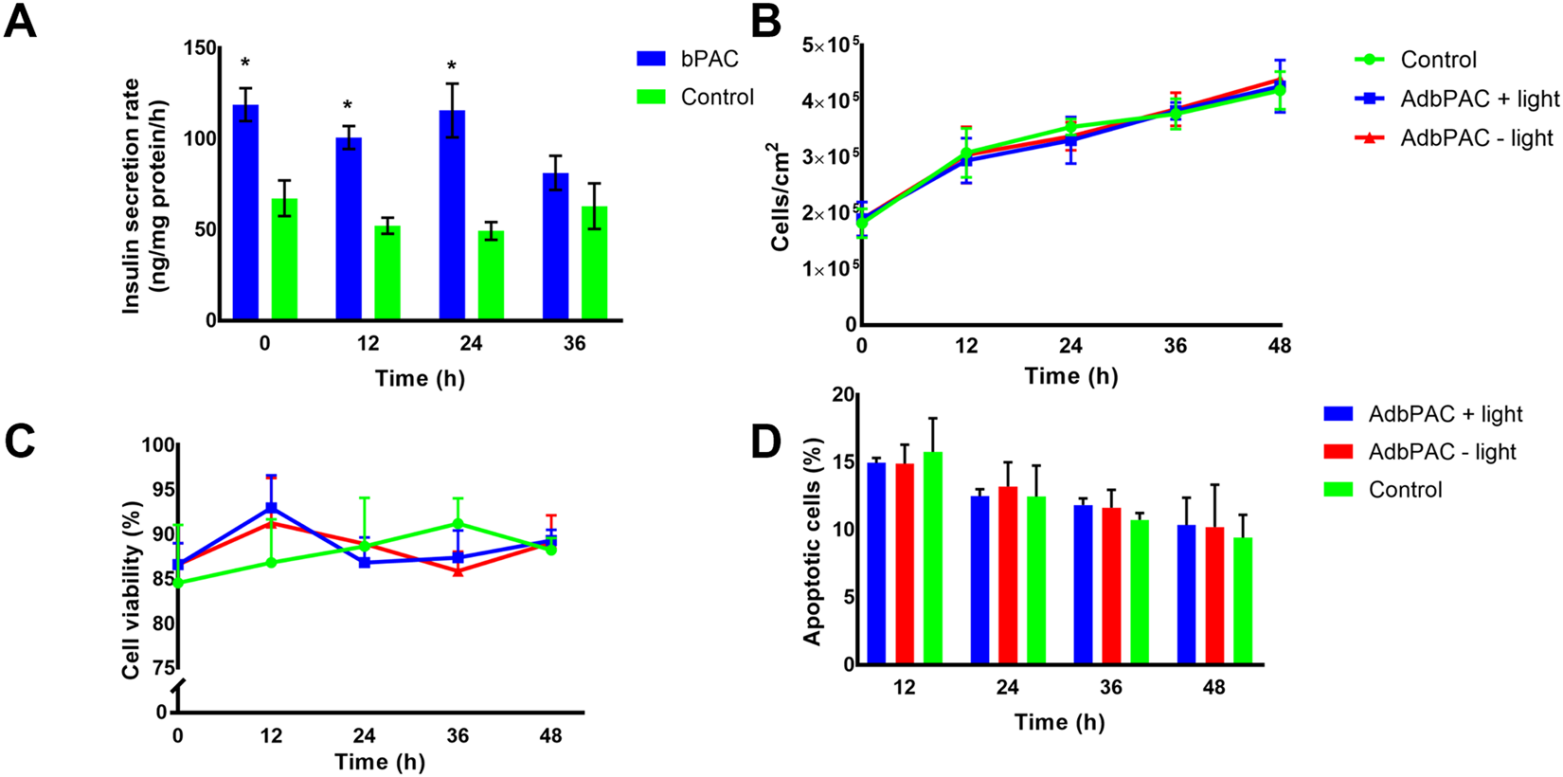
Repeated stimulation of β-cells expressing bPAC. (**A**) Cells were illuminated with blue light every 12 hours for 2 days. Cells expressing bPAC (bPAC) displayed higher rates of insulin release (ng insulin/mg total protein/h) upon exposure to light vs. non-transduced MIN6 β-cells (control). MOI = 100; *p < 0.05 vs. control cells. (**B**) Proliferation, (**C**) cell viability and (**D**) apoptosis (TUNEL) of MIN6 β-cells expressing bPAC and stimulated with light (blue curve), kept in dark (red curve) or non-transduced cells (green). The time points correspond to those shown in (**A**) plus a final time point (48 h) at 12 hours after the last stimulation for insulin secretion. Results are shown as mean ± SD from at least three independent experiments.

Transduced cells were also subjected to repeated cycles of light stimulation to assess the secretory capacity of β-cells over successive activations of bPAC and the potential effects on viability (Fig. 3A). Cells were stimulated with light every 12 h for 2 days. The rate of insulin release for bPAC-expressing cells exposed to light at 2.5 mM glucose remained constant and higher than for control cultures. After 36 h, insulin release was similar to that of the control cells most likely because of the ‘dilution effect’ caused by cell proliferation on the expressed transgene that was delivered with an adenoviral vector. Indeed, proliferation did not differ among non-transduced cells and bPAC-expressing cells stimulated with light or kept in the dark (Fig. 3B). Even the fractions of viable and apoptotic cells in these populations were similar after four repeated stimulations with blue light (Fig. 3C,D) suggesting that there were no adverse effects of cAMP modulation on the cell viability and proliferation.

### Light-triggered enhancement of insulin release in murine islets and MIN6 β-cell pseudoislets expressing bPAC

**Figure 4:**
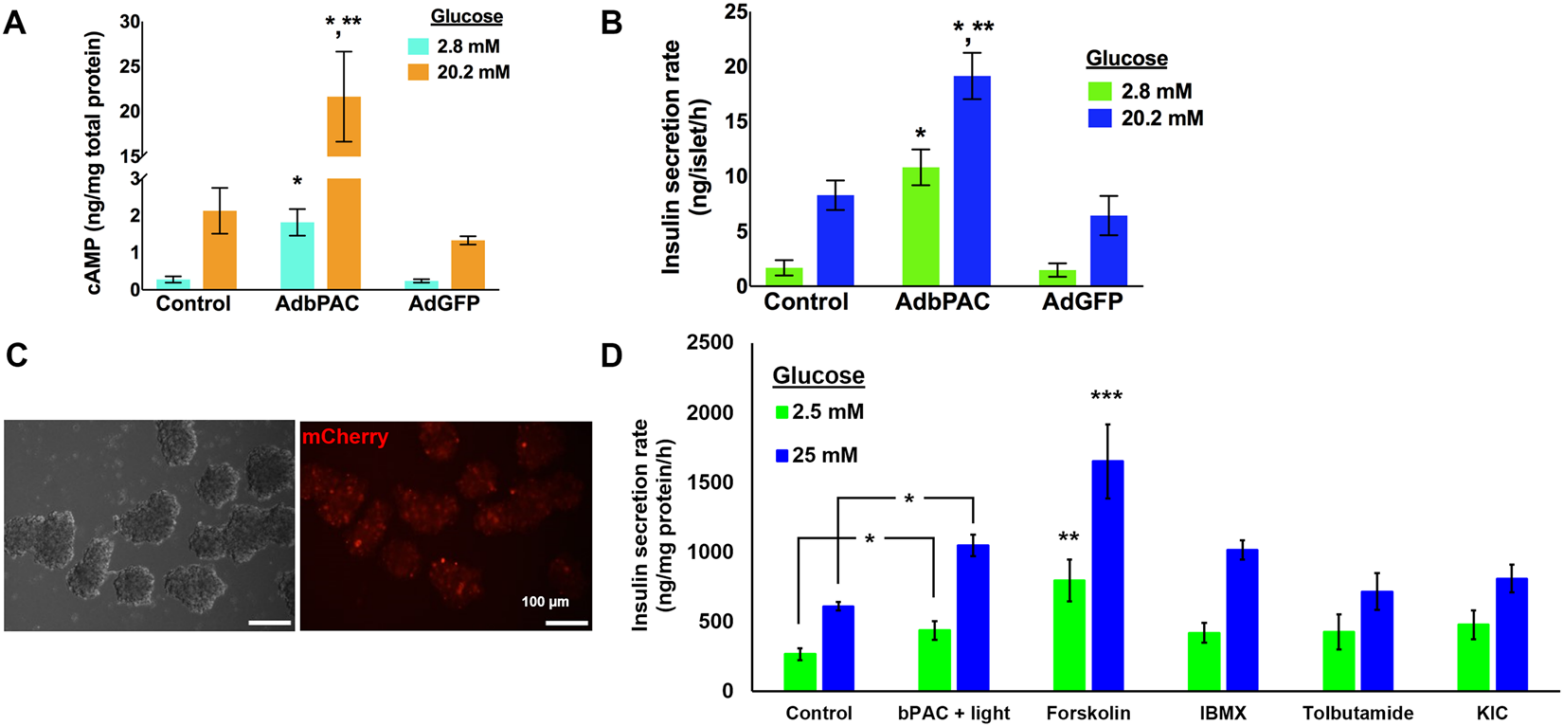
Expression of bPAC by murine islets and MIN6 β-cell PIs. (**A**) [cAMP]_i_ content and (**B**) insulin secretion rate of murine islets without transduction (control) or transduced with AdbPAC or AdGFP. All islets were incubated with the stated concentration of glucose while exposed to blue light. *p < 0.01 AdbPAC-transduced vs. control islets in corresponding conditions. **p < 0.01, AdbPAC-transduced islets at 20.2 mM vs. 2.8 mM glucose. (**C**) Micrographs of PIs of MIN6 β-cells transduced with AdbPAC. Brightfield and epifluorescence (mCherry) images are shown. Bar: 100 µm. (**D**) The rate of insulin release by PIs of MIN6 β-cells expressing bPAC was higher upon illumination (30 min) with blue light (bPAC + light; MOI = 100) compared to PIs of regular MIN6 cells (control). *p < 0.05. For forskolin treatment: **, ***p < 0.05 vs. control and bPAC + light at 2.5 mM or 25 mM glucose, respectively. Treatment with IBMX, tolbutamide or KIC resulted in significantly higher release rates than those of the control samples (control; p < 0.05) for the corresponding glucose concentration but similar to those of PIs expressing bPAC and exposed to light.

In addition to β-cells lines, which represent relatively homogeneous β-cell populations, we determined the effect of bPAC activation in murine islets. Islets expressing bPAC and exposed to blue light for 30 min exhibited higher [cAMP]_i_ both at 2.8 mM (1.82 ± 0.36 ng cAMP/mg total protein) and 20.2 mM (21.66 ± 5 ng cAMP/mg total protein) of glucose compared to islets not carrying the bPAC gene (0.28 ± 0.08 (2.8 mM) and 2.13 ± 0.62 (20.2 mM) ng cAMP/mg total protein; Fig. 4A). This translated to augmented insulin secretion for islets transduced with the AdbPAC (Fig. 4B). The insulin secretion rate of primary islets at 20.2 mM glucose was almost 5-fold higher than the corresponding rate at 2.8 mM glucose. This was not different (~4.5-fold) for islets transduced with AdGFP but was higher than for AdbPAC-expressing islets, which displayed greater insulin secretion rates both at 20.2 mM and 2.8 mM of glucose, i.e. 19.14 and 10.83 ng insulin/islet/h (p = 6.7 × 10^−3^, n = 3), respectively. The higher hormone release by AdbPAC-transduced islets vs. control islets at 2.8 mM was aligned with the higher [cAMP]_i_ (Fig. 4A) similar to our observations with MIN6 β-cells described above. These findings also underline the amplifying role of cAMP on insulin secretion in the presence of glucose.

Dispersed islet cells including primary and insulinoma β-cells re-organize into aggregates or ‘pseudoislets’ (PIs) exhibiting ultrastructural characteristics of native islets and higher GSIS^27, 28^ than monolayer cultures. We have shown that insulinoma PIs, which can be formed either in static or stirred-suspension cultures, respond to glucose and secretagogues with enhanced insulin secretion^29^. Thus, we questioned if this response could be further enhanced with photostimulation of PIs comprising β-cells expressing bPAC. To address this, MIN6 β-cells transduced with AdbPAC were cultured to form PIs (Fig. 4C). These PIs showed greater rates of insulin release upon illumination than PIs of control MIN6 β-cells both at 2.5 mM (439.32 ± 66.43 vs. 267.47 ± 41.05 ng insulin/mg total protein/h, p = 0.018, n = 3) and 25 mM of glucose (1048.82 ± 75.3 vs. 612.37 ± 31 ng insulin/mg total protein/h, p = 7.5 × 10^−4^, n = 3) (Fig. 4D). Of note, the ratio of rates at 25 mM over 2.5 mM of glucose was similar for PIs with (~2.4) or without (~2.3) the expression of bPAC analogous to our observations in monolayer cultures. The enhanced secretion of insulin at either glucose level by PIs of bPAC β-cells exposed to light was not different than that by PIs treated with IBMX, tolbutamide or KIC (Fig. 4D) but were lower compared to that of PIs incubated with forskolin (2.5 mM: 796.64 ± 148.92, p = 0.019; 25 mM: 1652.52 ± 264.88 ng insulin/mg total protein/h, p = 0.019, n = 3). Hence, insulin secretion can be modulated optogenetically in primary islets and β-cell PIs. We found in the latter that the enhancement achieved through blue light illumination is equivalent to that gained by treating PIs with known chemical secretagogues.

### Optogenetic regulation of insulin release is Ca^2+^-dependent

**Figure 5:**
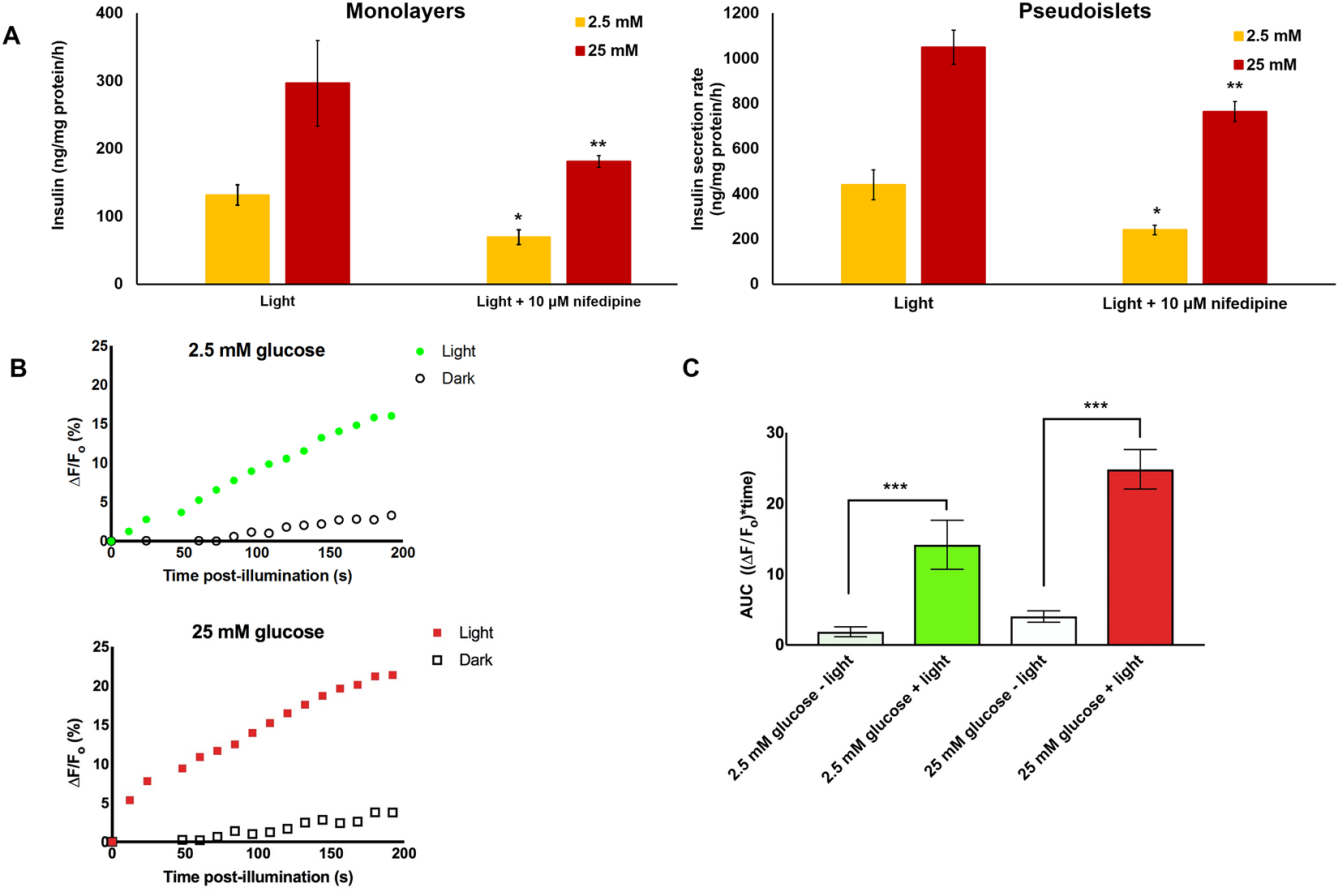
Optogenetic enhancement of insulin release in bPAC-expressing MIN6 β-cells is Ca^+2^-dependent. (**A**) Insulin secretion rates for AdbPAC-transduced MIN6 β-cells in monolayers (left graph) or PIs (right graph). Light: Cells were illuminated for 30 min. *, **p < 0.05 vs. corresponding samples exposed to blue light in the absence of nifedipine at 2.5 mM or 25 mM glucose, respectively. (**B**) Effect of blue light irradiation on the [Ca^2+^]_i_ of bPAC-expressing MIN6 β-cells exposed to 2.5 mM (left) or 25 mM glucose (right). The fluorescence intensities of irradiated (20 s; filled markers) and unirradiated cells (hollow markers) were measured for up to 200 s post-irradiation. The results are shown from representative experiments, as the ratio (percentage) of the change in fluorescence intensity from baseline (ΔF = F − F_o_) to the baseline intensity (F_o_). (**C**) As for (**B**) but presenting the area under the curve (AUC) from three independent experiments. ***p < 0.001.

The increase in β-cell [cAMP]_i_ due to extracellular glucose elicits events leading to plasma membrane depolarization and opening of the L-type voltage-dependent Ca^2+^ channels that mediate ion influx, subsequent fusion of secretory vesicles and the secretion of the hormone. In fact, agents (e.g. KCl) that elevate [Ca^2+^]_i_ in β-cells can trigger insulin secretion even in the absence of glucose. We thus wondered if the Ca^+2^ dependence is maintained during insulin secretion in our system given that photoactivation of bPAC induces an increase in [cAMP]_i_. Released insulin was measured in monolayer or PI cultures of bPAC-expressing cells upon incubation with nifedipine, a blocker of L-type Ca^+2^ channels (Fig. 5A). In monolayers, the inclusion of the Ca^2+^-channel blocker reduced the secretion rate by 47% and 40% at 2.5 and 25 mM glucose, respectively. Nifedipine treatment led to a similar decrease in PIs at 2.5 mM (45%) but the decline was less pronounced at 25 mM (27%). We also obtained evidence of the light-triggered changes in [Ca^2+^]_i_ by analyzing ion influx after incubation with CaSiR-1. A greater increase in [Ca^2+^]_i_ was noted in cells irradiated with blue light than in those cells kept in dark (Fig. 5B,C). Therefore, the insulin secretion is Ca^2+^-dependent in bPAC expressing-β-cells similar to native β-cells. Moreover, the blue light activation of bPAC results in [Ca^2+^]_i_ increase aligned with the augmented insulin release by these cells.

## Discussion

Exogenous regulation of [cAMP]_i_, which is an amplifier rather than an inducer of insulin secretion, is a promising avenue for enhancing β-cell function particularly for clinical applications. [cAMP]_i_ levels are dictated mainly by cAMP synthesis and degradation by ACs and PDEs, respectively. *In vivo* AC activation with incretins to elevate [cAMP]_i_ is hampered by their rapid clearance by dipeptidylpeptidase-4 (DPP4) and PDE inhibition is challenging because islet cells express multiple PDE types^30, 31^. Here, we describe the optogenetic regulation of [cAMP]_i_ and thus of insulin secretion in β-cells. To our knowledge, control of insulin release via optogenetic modulation of [cAMP]_i_ using a photoactivatable AC has not been demonstrated to date. In recent reports, ectopic expression of ChRs in MIN6 β-cells and islets led to changes in hormone secretion through light-triggered variations in Ca^2+^ influx^9, 10^. Interestingly, illumination induced the release of insulin by these cells in a manner that was independent of glucose metabolism. An almost 40% increase in insulin was noted after ChR stimulation in glucose-free medium^9^, and secretion was enhanced at low but not at high glucose concentrations^9, 10^. Moreover, ChRs require a ‘co-factor’ (retinal) for light-triggered activity, and the sequence of processes involving ChR-mediated [Ca^2+^]_i_ increase, activation of gene transcription (e.g. GLP-1^8^) and translation may result in a slower overall response. Furthermore, high-fat diet and fatty acids enhance [Ca^2+^]_i_^32, 33^ making plausible the hypothesis that an exaggerated Ca^2+^ response could contribute to the initial hyperinsulinemia seen in T2D. This could confound the prospective regulation of Ca^2+^ signaling as a way to potentiate insulin production. Hence, targeting cAMP directly may be advantageous because its increase either by treatment with agents such as forskolin and IBMX or through bPAC activation with light amplifies GSIS. We observed that illumination of bPAC-expressing β-cells (MIN6, βTC) and primary islets resulted in increased secreted insulin even at low glucose concentrations but not in glucose-free medium. The observed response is aligned with the role of cAMP as an amplifier of insulin release in the presence of glucose. Indeed, no change in basal insulin release by MIN6 cells was noted in buffer devoid of glucose despite a raise in [cAMP]_i_ due to bPAC activation. Therefore, this mode of amplification of the secretory function does not abolish control of glucose on insulin release in β-cells.

The augmented insulin production due to [cAMP]_i_ elevation also points to a reduced number of β-cells or islets required – for instance, in a transplantation setting – to generate sufficient hormone amounts for glucose homeostasis. In fact, the secretion rate of bPAC-expressing β-cell monolayers or PIs was enhanced evenly and proportionally at 2.5 mM and 25 mM maintaining approximately the same constant ratio (2.2–2.5) as regular MIN6 cell monolayers or PIs. Such even increase in insulin secretion was not observed in primary islets. This insulin secretion ratio was lower after photoactivation of bPAC (~1.8) in primary islets compared to non-transduced islets (~5). This can be attributed to the pronounced enhancement of insulin release at 2.8 mM glucose with bPAC activation. Other factors however, may also need to be factored in such as a potential upper limit in the release of insulin at high glucose despite the significantly elevated [cAMP]_i_ and the presence of other endocrine cells, which are also exposed to AdbPAC upon transduction of the islets.

When exposed to blue light for 30 minutes every 12 hours, AdbPAC-transduced MIN6 β-cells exhibited consistently higher insulin secretion over 36 hours but a slight decline was noted subsequently. Lower viability and enhanced apoptosis were ruled out as reasons for this decline. The reduction in insulin secretion, which was still on par with that from non-transduced MIN6 β-cells, was most likely due to a ‘dilution effect’ associated with the continuous cell proliferation and transient transgene expression. This issue can be addressed with the generation of β-cell lines stably expressing genes encoding for photoactivatable ACs.

The system also exhibited favorable kinetics with the [cAMP]_i_ increasing within 5 minutes of photoactivation. This timeframe is comparable with that of [cAMP]_i_ increase in murine β-cells within intact pancreatic islets following elevation of glucose concentration from 3 to 20 mM^24^. The [cAMP]_i_ returned to a stable level within 8 minutes after illumination was terminated but was marginally higher than the baseline, i.e. for cells kept in dark. This did not affect the proliferation and viability of the cells over multiple cycles of stimulation. In fact, elevated [cAMP]_i_ (e.g., after incretin stimulation) promotes β-cell proliferation through PKA-dependent mechanisms and suppresses apoptosis^34, 35^ partly due to the increased expression of the anti-apoptotic proteins Bcl-2 and Bcl-xL^36, 37^. Further studies are warranted about the potential long-term effects of optogenetic modulation of [cAMP]_i_ on the β-cells.

To that end, β-cells engineered to express bPAC can be used as a tool for studying the role of [cAMP]_i_ and its interaction with other signaling pathways in normal islets and those from diabetes patients. For instance, a bidirectional relation between [cAMP]_i_ and [Ca^2+^]_i_ fluctuations has been hypothesized although the exact mechanism remains unclear^38^. Also, maladaptation of cAMP signaling has been reported in several animal models of diabetes including decreased glucose-induced production of [cAMP]_i_^39^ and lower expression of PDEs^40^. *In vitro*, prolonged culture of β-cells in high concentrations of glucose results in alterations in [cAMP]_i_ activity^41^. Extensive β-cell juxtaposition seen in PIs increases cAMP responsiveness^42^ resulting in augmented GSIS compared to monolayer β-cell cultures as we and others have observed. These observations raise important questions, many of which can be potentially addressed using an optogenetic platform such as the one described here.

In addition to their value as a research tool, bPAC-expressing β-cells can be utilized, for example, as PIs, in a retrievable encapsulation device. This could be integrated into an autonomous bioartificial pancreas device with a low-power light source and existing glucose sensors (e.g. a wireless sensor fitted in a contact lens^43^) to control illumination. The blue light’s relatively poor tissue penetration is not expected to hinder the efficient stimulation of cells in this setup. Yet, the sensitivity of PACs can be shifted toward the infrared spectrum through protein engineering^44^. Additionally, available human β-cell lines^45, 46^ which are clinically more relevant than their rodent counterparts, can be modified for optogenetic regulation of cAMP. Beyond β-cell lines, human pluripotent stem cell (hPSC)-derived insulin-secreting cells may also be engineered to express PACs aiming at correcting defective GSIS, which is a major challenge in the generation of functional β-cells from hPSCs. Overall, optogenetic approaches such as the one presented here are expected to enable technologies which will enhance our understanding of β-cell physiology and contribute to biomedical solutions for pancreas pathologies.

## Materials and Methods

### Cell culture

Mouse MIN6^47^ and βTC^48^ β-cells were maintained in DMEM containing 10% fetal bovine serum (FBS) (all from ThermoFisher Scientific, Waltham, MA) at 37 °C and 5% CO_2_. Cells were split 1:4 at 70–90% confluence. PIs were formed by seeding 2–5 × 10^4^ cells/ml onto Petri dishes or 10^5^ cells/ml in 125-ml ProCulture spinner flasks^29^ (Corning, NY). In spinner flask PI cultures, half-volume medium changes were performed every 2–3 days. AD293 cells (Cell Biolabs Inc., San Diego, CA) used for recombinant adenovirus production were cultured in the same medium as above but supplemented with 1% MEM non-essential amino acids (ThermoFisher). Cell viability was determined by Trypan Blue staining (ThermoFisher) using a hemocytometer or a TC20 counter (Bio-Rad Laboratories, Hercules, CA).

### Murine islet isolation and culture

An abdominal incision was performed to 8-week old male C57BL/6 mice under anesthesia to access the pancreas, which was inflated and harvested. Digestion was carried out with collagenase (CIzyme RI from Vitacyte, Indianapolis, IN). Gradient centrifugation and gravity sedimentation of islets were carried out in lymphocyte separation medium (Corning) and Hank’s balanced salt solution (ThermoFisher). Islets were handpicked under a microscope, counted and cultured in RPMI 1640 (MP Biomedicals, Santa Ana, CA) supplied with 10% FBS (ThermoFisher) in Petri dishes at 37 °C and 5% CO_2_. Intact islets underwent two rounds of adenoviral transduction (MOI as stated, ~1,500 cells/islet) 48 h apart.

### bPAC gene and adenovirus construction

A human-codon optimized bPAC gene was synthesized (GeneArt, ThermoFisher) from the available bPAC sequence (accession number: GU461306.2)^21^. A cMyc-derived epitope (myc tag: EQKLISEEDL) was added to the C-terminus of bPAC followed by an internal ribosomal entry site (IRES) sequence, the mCherry gene and the SV40 polyA tail. The bPAC-cMyc-IRES-mCherry cassette was inserted into the pShuttle-CMV vector for generation of an adenovirus (AdbPAC) in 293AD cells with the AdEasy system (Agilent Technologies, Santa Clara, CA)^49^. The AdGFP adenovirus carrying the GFP gene under the CMV promoter was obtained from the Baylor College of Medicine Vector Development Core (Houston, TX). Purification and titer determination of the adenoviral particles were carried out using the ViraBind and QuickTiter kits (Cell Biolabs), respectively. For AdbPAC propagation, 293AD cells were transduced at 70% confluence.

### Arduino-driven LED arrays for blue light stimulation

Arrays were constructed with 6 or 12 LEDs (470 nm; Mouser Electronics, Mansfield, TX) for stimulation of cells cultured in 6- or 12-well plates. For control of its operation, each array was connected to an Arduino board (Adafruit, New York, NY) that was programmed accordingly. In this configuration, the light power on the culture surface was 0.18 mW/mm^2^ measured with a S120C sensor connected to PM100D light meter (Thorlabs Inc., Newton, NJ). For pulsed stimulation the light cycle was 5 sec on/10 sec off.

### [cAMP]_i_ and secreted insulin assays

[cAMP]_i_ and secreted insulin were determined typically 48 h after transduction. For experiments with murine islets, between 8–20 islets (per well of 24-well plate) were used for each condition. Medium was replaced with Krebs-Ringer bicarbonate buffer (KRB (mM): 129 NaCl; 4.8 KCl; 1.2 KH_2_PO_4_; 1.2 MgSO_4_; 2.5 CaCl_2_; 5 NaHCO_3_; 10 HEPES, pH = 7.2) with 0.1% BSA and 2.5 mM glucose. After 3 h, the buffer was replaced with fresh KRB with no glucose, 2.5 mM (cells/PIs) or 2.8 mM (murine islets) (basal level) and 25 mM (cells/PIs) or 20.2 mM (murine islets) glucose (supraphysiological level) as stated and cells/islets were further incubated with or without illumination. At the same time, the AC inhibitor MDL12330A^50^ (100 μM; Sigma-Aldrich, St. Louis, MO), L-type Ca^2+^ blocker nifedipine (10 µM; Abcam, Cambridge, MA) or secretagogues (all from Sigma-Aldrich) were also added as stated: 10 µM forskolin, 10 µM 3-isobutyl-1-methylxanthine (IBMX), 500 nM phorbol 12-myristate 13-acetate (PMA), 100 µM tolbutamide and 15 mM ketoisocaproate (KIC). Cells were lysed at different times in 0.1 M HCl for determination of [cAMP]_i_ via enzyme-linked immunosorbent assay (ELISA; Cayman Chemical Co., Ann Arbor, MI) following the manufacturer’s instructions. Supernatant was collected and analyzed for secreted insulin by ELISA (EMD Millipore, Billerica, MA) as described^29^. Insulin and [cAMP]_i_ measurements were normalized with the total protein content of the corresponding cell lysates determined via the Bradford method (ThermoFisher). Of note, insulin secretion for primary islets was normalized with respect to the number of islets utilized.

Experiments involving multiple cycles of stimulation with light, were initiated 48 h post-transduction. For this purpose, the following procedure was repeated every 12 h: The culture medium in all wells of 12-well plates was replaced with KRB containing 2.5 mM glucose and cells were incubated for 2 h. Then, KRB was removed and fresh KRB with 2.5 mM glucose was added for 30 min along with or without blue light illumination. Subsequently, supernatants and cells were collected from at least 3 wells for determination of secreted insulin and total protein (as described above). The buffer in the remaining wells was replaced with fresh culture medium and the procedure was repeated 12 h later for up to 2 days.

In these experiments, cell numbers and the fractions of live (Trypan Blue; see above) and apoptotic cells (TUNEL assay; see below) were determined from samples collected before the exchange of medium with KRB.

### TUNEL assay

DNA fragmentation-related apoptosis was assessed by terminal deoxynucleotidyl transferase dUTP nick-end labeling (TUNEL) using the FlowTACS apoptosis detection kit (Trevigen) according to the manufacturer’s instructions as we reported^29^. Briefly, cells in aliquots were fixed with 3.7% formaldehyde for 10 min and stained with 10 μg/ml propidium iodide in PBS for 5 min. Then, the samples were permeabilized with cytonin for 30 min, labeled with terminal deoxynucleotidyl transferase (TdT) for 40 min at 37 °C and incubated with a streptavidin–FITC antibody in the dark for 10 min. After two washes with PBS, the samples were analyzed via flow cytometry. Appropriate gating was applied to screen out cellular debris and cell clumps. Positive (cells treated with nuclease) and negative (cells without TdT primary antibody incubation) controls were used to set up bins for apoptotic, and non-apoptotic ranges.

### Immunocytochemistry

Cells were fixed with 4% paraformaldehyde (Sigma) in PBS for 20 min, permeabilized/blocked in PBS with 0.1% Triton X-100 (Mallinckrodt Baker, Phillipsburg, NJ) and 1% bovine serum albumin (BSA; Sigma) for 30 min and incubated overnight at 4 °C with a rabbit antibody against the myc epitope (cat. no. 2272, Cell Signaling Technology, Danvers, MA). After three washes with PBS, cells were incubated with a donkey anti-rabbit secondary antibody conjugated to Alexa 488 (Jackson Immunoresearch Laboratories Inc., West Grove, PA) for 1 h at room temperature. Nuclear DNA was stained with DAPI (Vectashield, Vector Laboratories, Burlingame, CA). Images were acquired with a Leica TCS SPE confocal microscope (Leica Microsystems Inc., Buffalo Grove, IL) and quantitative analysis was performed with the Leica LAS X software. Images from at least 10 separate fields per experiment were used for analysis.

### Western blot analysis

Cells were lysed in radioimmunoprecipitation (RIPA) buffer supplemented with a protease inhibitor cocktail (EMD Biosciences, CA) for 30 min and total protein was determined (Bradford; ThermoFisher). Cell lysates were boiled for 5 min at 95 °C, loaded to a sodium dodecyl sulfate-polyacrylamide gel (30 μg of total protein/lane) and after gel electrophoresis and protein transfer to polyvinylidene difluoride (PVDF) membranes (Thermo Fisher Scientific), the membranes were blocked with 5% milk in Tris-buffered saline with 0.05% Tween-20 (TBS/T) for 1 h at room temperature. After 3 washes with TBS/T, the membranes were incubated with primary antibodies against myc-tag (Cell Signaling Technology) and β-actin (cat. no. A1978, Sigma) in a shaker at 4 °C overnight. Following 3 more TBS/T washes, secondary horseradish peroxidase (HRP)-conjugated antibodies (Jackson ImmunoResearch Laboratories Inc.) were added for 1 h at room temperature. Membranes were washed 3 times and proteins were detected in a C-DiGit blot scanner after adding WesternSure ECL substrate (Li-Cor Biotechnology, Lincoln, NE).

### Intracellular Ca^2+^ measurement

Cells were washed once with Hank’s Balanced Salt Solution (HBSS) and loaded with the calcium indicator CaSiR-1 acetoxymethyl ester (Goryo Chemical Inc., Sapporo, Japan) (5 μM final concentration) at 37 °C for 20 min in the dark^51^ according the manufacturer’s instructions. The loading solution was then replaced with KRB containing 2.5 mM or 25 mM glucose. Cells were placed on a Leica TCS SPE confocal microscope (Leica Microsystems Inc., Buffalo Grove, IL) and irradiated with a 488-nm laser light for 20 s immediately followed by the recording of fluorescence intensity in the range of 650–750 nm (emitted light) after excitation with a far-red (635 nm) laser light. Cells in each field were selected based on mCherry fluorescence (excitation with a 532-nm laser) and analyzed using the Leica LAS X software. Between 20–30 cells were analyzed per sample. A region of interest was defined for each cell and the average fluorescent intensity was calculated. These intensities were averaged over all mCherry-positive cells. Average intracellular Ca^2+^ levels were quantified as the change in average fluorescence intensity at different time points above the initial fluorescence (ΔF = F − F_o_) divided by the initial intensity (F_o_). The area under the curve (AUC) was calculated from temporal data of ΔF/F_o_ via a script in MATLAB (Mathworks, Natick, MA).

### Statistical analysis

Results are expressed as mean ± standard deviation (SD) from at least 3 independent experiments with each experimental point analyzed in triplicates. Analysis of variance and the *posthoc* Tukey test were performed using Prism 7 (GraphPad Software, Inc., La Jolla, CA). Values of p < 0.05 were considered as significant.

## Electronic supplementary material

Supplementary Information
