# Supplementary material for: Optogenetic regulation of insulin secretion in pancreatic β-cells
Source: Sci Rep. 2017 Aug 24;7:9357. doi: 10.1038/s41598-017-09937-0 (PMC5571193; doi:10.1038/s41598-017-09937-0)
Supplement: Supplementary file 1 — Supplementary Information [file 41598_2017_9937_MOESM1_ESM.pdf]

Optogenetic regulation of insulin secretion in pancreatic β-cells

Fan Zhang[1]*, BS, E. S. Tzanakakis*[1,2*]*, PhD*

[1]Department of Chemical and Biological Engineering, Tufts University, Medford, MA 02155
[2]Clinical and Translational Science Institute, Tufts Medical Center, Boston, MA 02111

- Fan Zhang, Graduate Student, Chemical and Biological Engineering, Tufts University, Medford, MA, 02155, Fan.Zhang@tufts.edu
- Emmanuel S. Tzanakakis, Associate Professor, Chemical and Biological Engineering, Tufts University, Medford, MA, 02155, Emmanuel.Tzanakakis@tufts.edu

[*]Corresponding author:

Emmanuel S. Tzanakakis

Associate Professor

Department of Chemical and Biological Engineering

Tufts University

Science and Technology Center, Room 276A

Medford, MA 02155 USA

Phone: +1-617-627-0831

Fax: +1-617-627-3991

E-mail address: Emmanuel.Tzanakakis@tufts.edu

Suppl. Fig. S1.

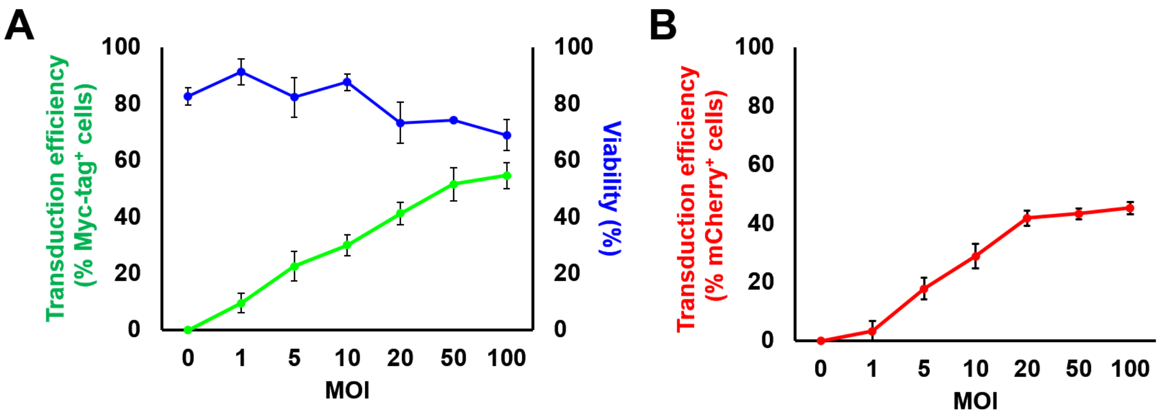

Figure S1. Transduction efficiency and viability. MIN6 β-cells were infected with AdbPAC at different multiplicities of infection (MOI) with AdbPAC. Transduction efficiency was determined by (A) immunostaining and microscopy for the fraction of myc-tag-positive cells (the total number of cells was determined by staining the cell nuclei with DAPI), or by (B) flow cytometry from the fraction of cells expressing mCherry. Viability was estimated based on Trypan Blue dye exclusion (see Materials and Methods). Results are shown as mean±SD from three independent experiments. For each MOI (A) images were analyzed from 10 fields; (B) triplicates were analyzed.

Suppl. Fig. S2.

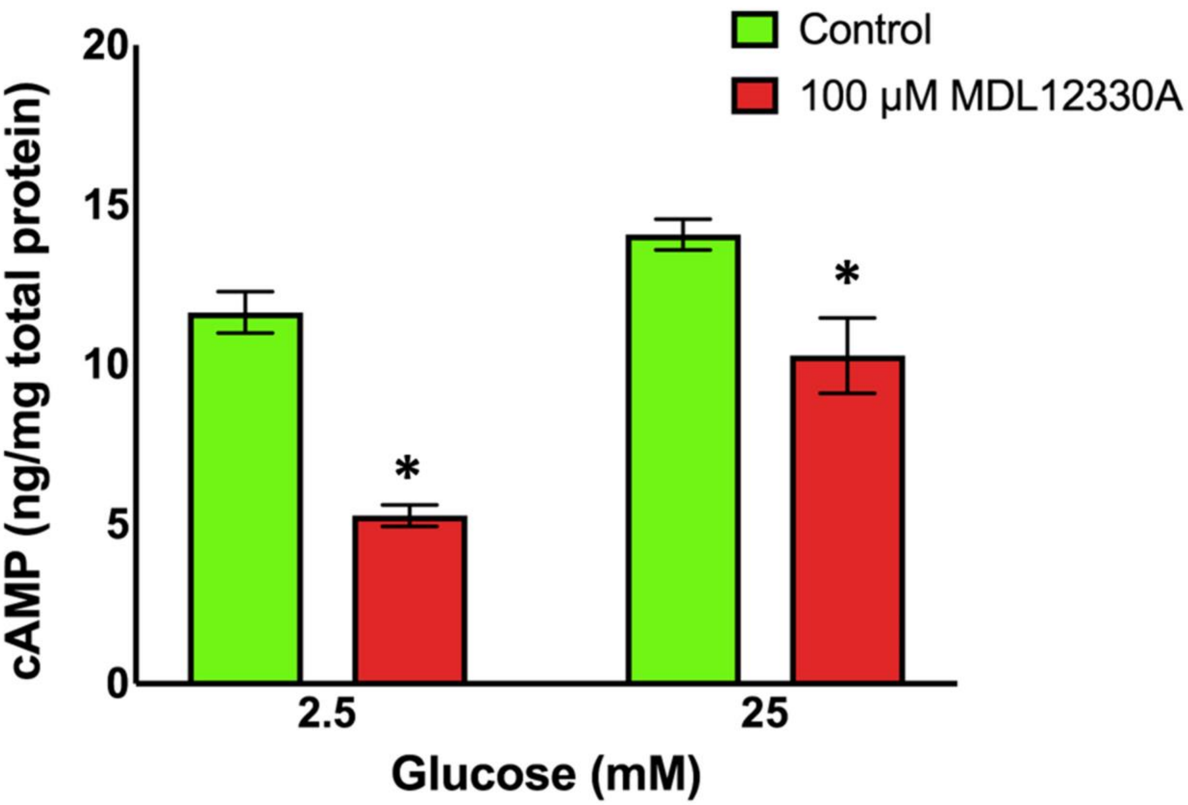

Figure S2. $[cAMP]_i$ by MIN6 β-cells expressing bPAC. Cells were infected with AdbPAC at MOI=100 and exposed to blue light for 30 min with or without (control) the AC inhibitor MDL12330A. Results are shown as mean±SD from at least three independent experiments. *$p<0.01$ vs. control cells.

Suppl. Fig. S3.

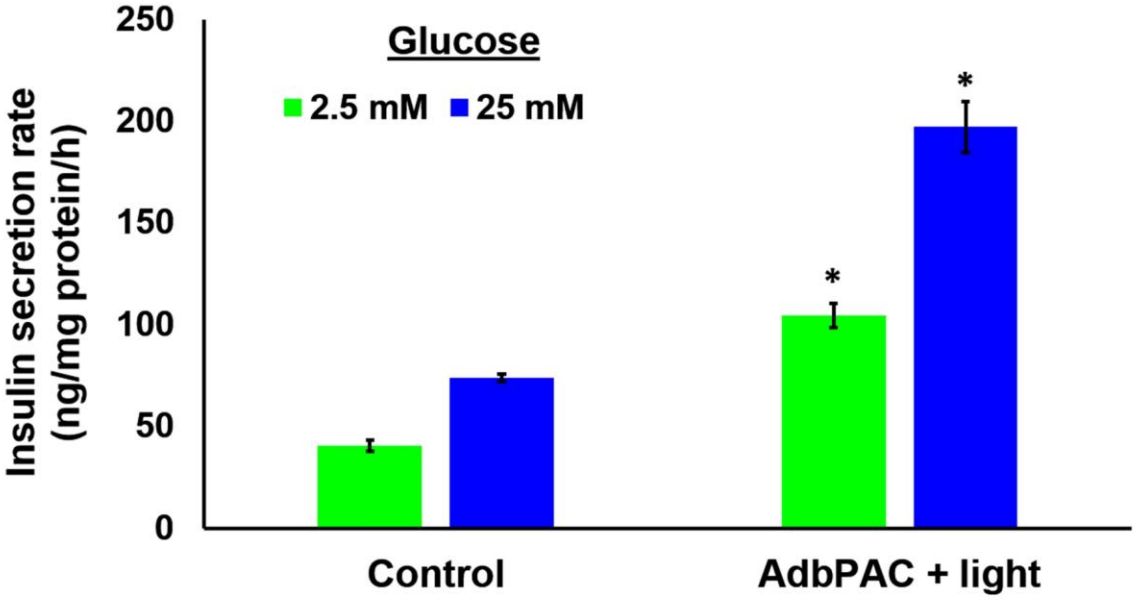

Figure S3. Insulin secretion by βTC cells expressing bPAC. Cells were infected with AdbPAC at MOI=100 and irradiated with light (AdbPAC + light). Results are shown as mean±SD from 3-4 independent experiments. *$p<0.05$ vs. corresponding cells without illumination (control).

Suppl. Fig. S4.

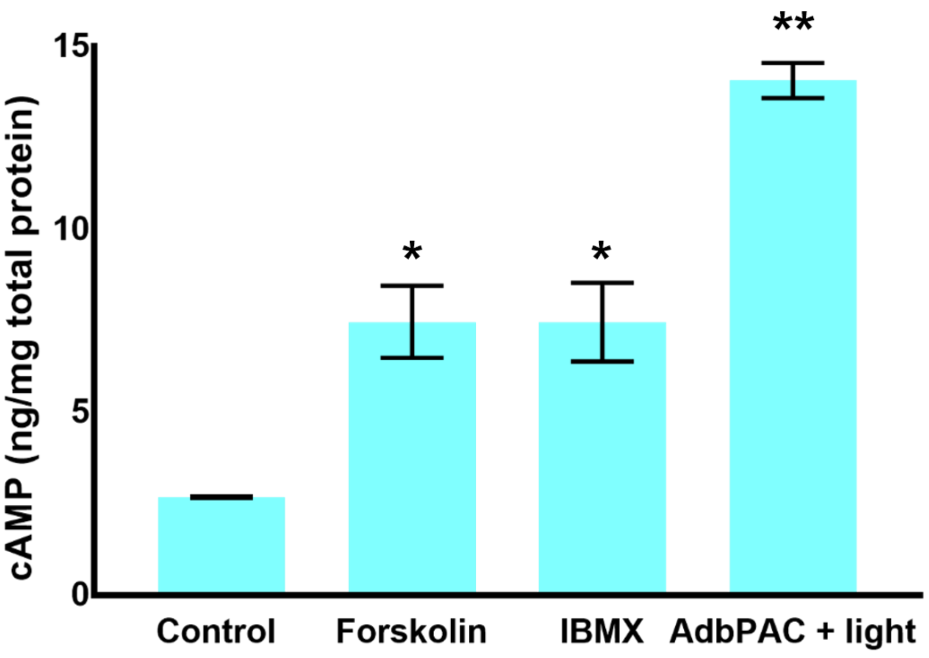

Figure S4. Levels of $[cAMP]_i$ in MIN6 β-cells expressing AdbPAC (MOI=100) and exposed to light (AdbPAC+light) or MIN6 β-cells treated with forskolin or IBMX. *$p<0.05$ vs. control cells (without transduction), **$p<0.05$ vs. forskolin- and IBMX-treated cells. Results are shown as mean±SD (n=3) from cells assayed in 25 mM glucose.

Supplementary Information. Some blots in Figure 1B have been edited for better (clear cut) presentation. The original blots are provided here.

Figure 1B – bPAC (myg tag):

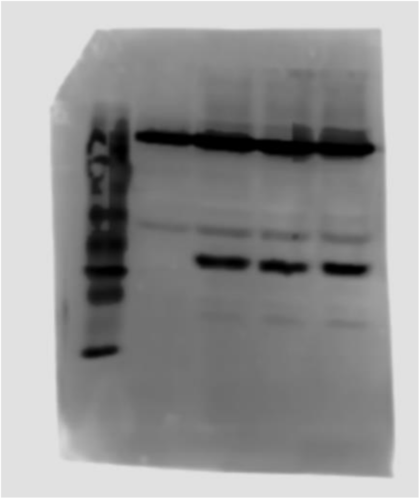

The lanes from left to right are: (1) Protein ladder, (2) non-transduced MIN6 β-cells, and MIN6 β-cells transduced with AdbPAC at an MOI of (3) 50, (4) 100, and (5) 200. The bPAC protein (myc tag) is detected at its estimated molecular weight of ~41 kDa. There are non-specific bands of higher molecular weight appearing in all samples (lanes 2-5).

Figure 1B – β-actin:

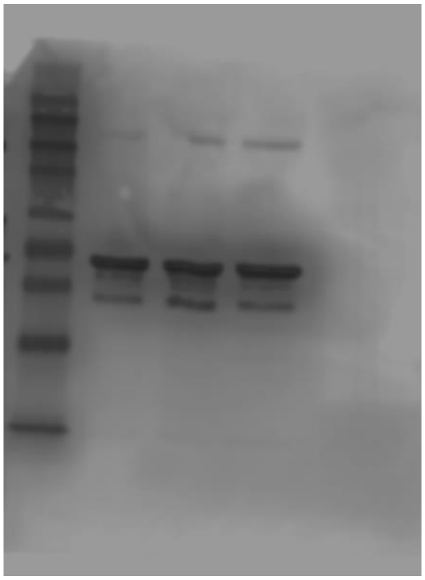

The same samples were run as in the blot for bPAC above except for the sample of cells infected with AdbPAC at MOI of 200. The lanes from left to right are: (1) Protein ladder, (2) non-transduced MIN6 cells, and MIN6 β-cells transduced with AdbPAC at an MOI of (3) 50, (4) 100. The expected band size is 45 kDa.
